# A Prevalence Estimation of Exstrophy and Epispadias in Germany From Public Health Insurance Data

**DOI:** 10.3389/fped.2021.648414

**Published:** 2021-10-26

**Authors:** Anne-Karoline Ebert, Nadine Zwink, Heiko Martin Reutter, Ekkehart Jenetzky

**Affiliations:** ^1^Department of Pediatric Urology, University Medical Center Ulm, University Hospital for Urology and Pediatric Urology, Ulm, Germany; ^2^Department of Child and Adolescent Psychiatry, University Medical Center of the Johannes Gutenberg University Mainz, Mainz, Germany; ^3^Department of Neonatology and Pediatric Intensive Care, University Hospital Erlangen, Erlangen, Germany; ^4^Department of Neonatology and Paediatric Intensive Care, Institute of Human Genetics, University Hospital Bonn, Bonn, Germany; ^5^Faculty of Health, School of Medicine, University of Witten/Herdecke, Witten, Germany

**Keywords:** prevalence, epispadias, bladder exstrophy epispadias complex (EEC), health insurance, public health, bladder exstrophy

## Abstract

**Introduction:** The prevalence of rare diseases is very important for health care research. According to the European Surveillance of Congenital Anomalies (EUROCAT) registers, the live prevalence for exstrophy and/or epispadias (grades 1–3) is reported with 1:23,255 (95% CI: 1:26,316; 1:20,000). A Europe-wide prevalence evaluation based on reports from excellence centers estimates a prevalence for exstrophies of 1:32,200 and for isolated epispadias of 1:96,800 in 2010. However, the frequency of exstrophy [International Statistical Classification of Diseases and Related Health Problems revision 10 (ICD-10): Q64.1] and epispadias (ICD-10: Q64.0) treated in different age groups in Germany remains unclear.

**Material and Method:** Public health insurance data from 71 million people (approximately 87% of the population) were provided by the German Institute for Medical Documentation and Information (DIMDI) in accordance to the German Social Insurance Code for this research purpose. DIMDI analyzed the data source for the ICD diagnoses exstrophy and epispadias between 2009 and 2011. As provided data were robust over the years, averaged data are mentioned. Detailed subgroup analysis of small numbers was forbidden due to privacy protection.

**Results:** Annually, 126 persons of all ages with epispadias and 244 with exstrophy are treated as inpatients. In the observed population, 34 infants (<1 year of age) with epispadias and 19 with exstrophy (58% male) are treated as outpatients each year. This corresponds to an estimated live prevalence of 1:11,000 (95% CI: 1:14,700; 1:8,400) for EEC (exstrophy–epispadias complex), more specifically a prevalence of 1:17,142 for epispadias and of 1:30,675 for exstrophy. The male-to-female ratio for exstrophy is 1.4:1 for infants and 1.6:1 for all minors. In children and adolescents, 349 epispadias and 393 exstrophies (up to the age of 17) are treated annually, whereas adults with exstrophy and even more with epispadias make comparatively less use of medical care.

**Conclusion:** With the help of DIMDI data, the live prevalence of bladder exstrophy and epispadias in Germany could be estimated. The prevalence of epispadias was higher than in previous reports, in which milder epispadias phenotypes (grade 1 or 2) may not have been included. These analyses might enlighten knowledge about nationwide incidence and treatment numbers of rare diseases such as the EEC.

## Introduction

The bladder exstrophy–epispadias complex (EEC) is one of the most serious congenital midline defects in humans. Today, the EEC is understood as a spectrum from an isolated genito-urethral defect affecting males and females (epispadias), to classical bladder exstrophy with an open bladder and pelvis in addition to the genital defect culminating in a multiorgan anomaly—the cloacal exstrophy. As it is shown in various recent long-term outcome studies, comparatively little substantial improvements in functional outcomes were achieved (1, 2). Although a rare disease, EEC treatment in Germany is decentralized with possible profound long-term sequelae such as unfavorable outcome related to small hospital treatment numbers (3, 4). A central aspect here remains that robust data regarding the incidence and live prevalence of EEC are still lacking. Although in 2009 a network for congenital uro-rectal malformations (CURE-Net) was established in Germany and a nationwide and centralized data collection about newborns with EEC was formed, obtained data remain incomplete as reporting was voluntary. The only EEC prevalence study in Germany estimated live prevalence from EEC reported cases through excellence centers (5). Still, prenatal terminated pregnancy rates of EEC affected individuals are not easily detectable. There exist some estimates from European Surveillance of Congenital Anomalies (EUROCAT) registries (6, 7). The incidence of individual malformation phenotypes may vary to an unknown extent across the spectrum of EEC (8). Accordingly, the number of children born in Germany per year or living individuals with EEC remains unclear.

As a consequence, scientific conclusions of clinical research remain limited in respect to their validity. Furthermore, the unmet need for more intensified and specific professional care for individuals with EEC of all ages cannot be structured, and patient-centered health system planning cannot be established. Incidence and prevalence data might put officials in the position, like in other rare diseases and other European countries, to bundle both professional and economic resources and thus might substantially improve the health care situation of people with EEC of all ages in Germany.

As an initial approach, free accessible codes of the operation and procedure classification system (OPS) from German general hospital sources were analyzed. However, in contrast to anorectal malformation (9), EEC relevant procedure codes were not unique. Recently, a legally guaranteed access to public insurance data in Germany was made available for scientific purposes. This covers the vast majority of the population; only high incomes have private insurances. Due to the great lack of reliable knowledge of the scientific community about the true numbers of EEC cases in Germany, the CURE-Net scientific consortium established this request for an anonymized analysis of unique data of EEC patients with public health insurance for the first three available years 2009, 2010, and 2011. The German Institute for Medical Documentation and Information (DIMDI) was assigned to a data source investigation to analyze the individual EEC patients according to the exact ICD-10 (International Statistical Classification of Diseases and Related Health Problems revision 10) diagnosis Q64.1 (bladder exstrophy) and Q64.0 (epispadias) from the years 2009 to 2011.

The purpose of this study is to evaluate the live prevalence of the EEC in Germany, to assess the male-to-female ratio, and to consider the treatment incidence of various age groups with the help of the German insurance documentation, including a representative nationwide population. Furthermore, the results of this methodology will be compared with further prevalence estimations.

## Materials and Methods

In the German private and public health insurance system, each patient served is categorized according to ICD-10 diagnoses, such as exstrophy (Q64.1), epispadias (Q64.0), cloaca (Q43.7), and possibly including cloaca exstrophy. The diagnoses serve for financial settlement for ambulatory and inpatients bills with a plausibility check. Although it is not specified within the ICD code clinically, epispadias can be differentiated according to the meatus location in glandular (grade 1), penile (grade 2), or penopubic (grade 3) (10). Similarly, as there is no specific ICD code for cloaca exstrophy, ICD code Q43.7 includes cloacal malformations as well; however, data for cloaca codes were also requested in spite of limited tolerable conclusions.

These health care data are provided by the DIMDI institute as part of the Federal Ministry of Health (BMG) and operates the “Information System Health Care Data” on its behalf in the course of the Data Transparency Ordinance. The request of data evaluation via a direct access is strongly limited to authorized institutions, such as universities, for research purposes and was allowed from 2009 onwards. Data of the public health insurances cover approximately 87% of the German population which is rated here to median 71,014,351 people (11). The request was written with a pre-specified Structured Query Language (SQL) script to be analyzed and prepared after application against payment by the “Data Preparation Office” at DIMDI. DIMDI provides an analysis of the so-called “DaTraV data” according to §303d of the Social security code five (SGB) V. As only anonymized data such as numbers of diagnoses of ambulatory and stationary patients a year were retrospectively made available from the DIMDI institute to the authors, no ethical approval is needed according to German law.

The data sets for three years (2009–2011) were provided and divided into three age strata for outpatient data and without age strata for inpatients. Additionally, specific case numbers of three requested ICD-10 codes related to EEC such as Q64.0, Q64.1, and Q43.7 were allocated. There is a statement from DIMDI that in their reporting each case is only counted once, meaning that no double cases are included. Due to data protection reasons, some detailed analyses were not allowed in subgroups of diagnosis, sex, and age group because of extremely small case numbers (<5). Due to the expected patient numbers in the case of rare diseases, the standard limit of 30 cases per group had already been reduced. Nevertheless, we could not obtain all favored sex and age strata. We received limited strata for three age levels: below 1 year of age, 1–17 years, and adults 18 years onwards. The data of approximately 71 million German insurance cases (*n* = 71,014,351) each year were analyzed over three consecutive years, corresponding to approximately 87% of the complete German population. This rate is calculated from the reported denominator data below 1 year and the published birth rate of the corresponding years: 582,832/668,586 of average births 2009–2011. The denominator in the estimation of live prevalence are nationwide public available newborns at the Federal Statistical Office (11). The first number is the average reported number of insurance cases below 1 year of age for the years 2009, 2010, and 2011. These numerators are used as the denominator for EEC rates as shown in all tables.

## Results

The inpatient data showed that each year generally 370 inpatients with public insurance were treated with bladder exstrophy or any epispadias in Germany from 2009 to 2011 (Table 1). According to their phenotype an average of 126 persons with public insurance had epispadias (range 101–144), and 244 had bladder exstrophy (range 216–260) each year. Since 87% of the population has statuary insurance, we estimate that approximately a total of 425 patients were treated each year. Due to very low case numbers and therefore data security reasons, differentiation between gender and age groups was not possible. The very rare cloaca malformation (Q43.7), which may include cases of cloacal exstrophy, was reported on average with 64 individuals per year (range 55–80) in an astonishingly high number in comparison to the bladder exstrophy cases.

**Table 1:** Inpatients with EEC, and due to small cell numbers no age and sex stratification.

**Type of malformation (ICD-10 code)**	**Basic population (0–99 years) of insurance sample (denominator)**	**Epispadias (Q64.0)^a^**	**Bladder Exstrophy (Q64.1)^a^**	**Cloaca (Q43.7) (which may include cloaca exstrophy)^a^**
Year 2009	71,059,552	133	260	55
Year 2010	70,958,660	144	257	57
Year 2011	71,024,842	101	216	80
Average in years 2009–2011	71,014,351	126 each year	244 each year	64 each year

a *Due to N <5 in stratified cells, no sex or age data are given*.

Outpatient data of adults (individuals 18 years onwards) indicated that 362 adults (range 351–371) with epispadias and 743 adults (range 736–747) with exstrophy were treated in the ambulatory setting. The male-to-female ratio was available only for exstrophy, showing a quite stable distribution with a male predominance of 1.322 (range 1.247–1.394) (Table 2) in adult treatment.

**Table 2:** Outpatient data of adult (18 years onwards) EEC patients.

**Type of malformation (ICD-10 code)**	**Year 2009**	**Year 2010**	**Year 2011**	**Average in years 2009–2011**
Basic adult (>17 years) of insurance sample (denominator)	T 59,258,500 = M 27,465,227 + F 31,793,273 Ratio: 0.864^a^	T 59,296,294 = M 27,508,664 + F 31,787,630Ratio: 0.865^a^	T 59,467,858 = M 27,633,780 + F 31,834,078 Ratio: 0.868^a^	T 59,340,884 = M 27,535,890 + F 31,804,994Ratio: 0.866^a^
Epispadias^b^ (Q64.0)	T 351 P 1:168,828	T 363P 1:163,351	T 371P 1:160,291	T 362P 1:163,925
Exstrophy (Q64.1)	T 747 = M 435 + F 312 Ratio: 1.394P 1:79,329^a^	T 746 = M 414 + F 332Ratio: 1.247P 1:79,486^a^	T 736 = M 420 + F 316 Ratio: 1.329P 1:80,799^a^	T 743 = M 423 + F 320Ratio: 1.322P 1:79,867^a^
Cloaca (Q43.7)^c^	T 108P 1:657,959	T 102P 1:695,673	T 126P 1:563,689	T 112P 1:634,057

*T, total, i.e., sum of female and male cases; F, female; M, male; Ratio, male-to-female ratio; P, live prevalence*.

a *Probably biased due to death rate, coding, or new detection*.

b *No sex strata*.

c *Cells below 5, hence no presentation for any age strata or sex*.

In the group of children and adolescents from 1 to 17 years, 315 (range 313–318) epispadias and 374 (range 367–379) exstrophies are treated annually in outpatient care, i.e., an average of 18.5 epispadias and 22 exstrophies per year (together 1:16,097). The male-to-female ratio for exstrophy is 1.597 for all minors with a range between the years from 1.561 to 1.681 (Table 3).

**Table 3:** Outpatient data of children and adolescents 1–17 years of age with EEC.

**Type of malformation (ICD-10 code)**	**Year 2009**	**Year 2010**	**Year 2011**	**Average in years 2009–2011**
Basic population (1–17 years) of insurance sample (denominator)	T 11,226,731 = M 5,757,645 + F 5,469,086 Ratio: 1.053	T 11,070,155 = M 5,678,681 + F 5,391,474Ratio: 1.053	T 10,975,021 = M 5,630,061 + F 5,344,960 Ratio: 1.053	T 11,090,636 = M 5,688,796 + F 5,401,840Ratio: 1.053
Epispadias^a^ (Q64.0)	T 313P 1:35,868	T 314P 1:35,255	T 318P 1:34,513	T 315P 1:35,208
Exstrophy (Q64.1)	T 378 = M 237 + F 141Ratio: 1.681P 1:29,700	T 379 = M 231 + F 148Ratio: 1.561P 1:29,209	T 365 = M 223 + F 142Ratio: 1.570P 1:30,069	T 374 = M 230 + F 144Ratio: 1.597P 1:29,654
Cloaca (Q43.7)^b^	T 108P 1:657,959	T 102P 1:695,673	T 126P 1:563,689	T 112P 1:634,057

*T, total, i.e., sum of female and male cases; F, female; M, male; Ratio, male-to-female ratio; P, live prevalence*.

a *No sex strata*.

b *Cells below 5, hence no presentation for any age strata or sex*.

In the group below 1 year of age, each year on average 34 infants with epispadias (i.e., 39 Germany-wide; 1:17,142) and 19 with exstrophy (11 of them male; 58%; i.e., 22 Germany-wide; 1:30,675) are treated at least once as outpatients (Table 4). These data likely represent the most valid prevalence estimation considering the need for surgery during this period. Among the infant group, exstrophy was noted in one out of every 27,166 males and one out of every 35,501 females. This corresponds to an estimated live prevalence for the complete EEC of 1:10,997 (95% CI: 1:14,700; 1:8,400).

**Table 4:** Outpatients below 1 year of age with EEC malformation for live prevalence estimation.

**Type of malformation (ICD-10 code)**	**Year 2009**	**Year 2010**	**Year 2011**	**Average in years 2009–2011**
Total birth rate in Germany	T 665,126 = M 341,249 + F 323,877Ratio: 1,054	T 677,947 = M 347,237 + F 330,710Ratio: 1,050	T 662,685 = M 339,899 + F 322,786Ratio: 1,053	T 668,586 = M 342,795 +F 325,791Ratio: 1,052
Birth rate (<1 year) of insurance sample (denominator) i.e., ~87.2%	T 574,321 = M 294,727 + F 279,594Ratio: 1.054	T 592,211 = M 303,306 + F 288,905Ratio: 1.050	T 581,963 = M 298,430 + F 283,533Ratio: 1.053	T 582,832 = M 298,821 +F 284,011Ratio: 1.052
Epispadias^a^ (Q64.0)	T 35P 1:16,409	T 37P 1:16,006	T 30P 1:19,399	T 34P 1:17,142
Exstrophy (Q64.1)	T 22 = M 11 + F 11Ratio: 1.0P 1:26,106	T 19 = M 11 + F 8Ratio: 1.4P 1:31,169	T 15 = M 10 + F 5Ratio: 2.0P 1:38,798	T 19 = M 11 + F 8Ratio: 1.4P 1:30,675
Cloaca (Q43.7)^b^	T 108P 1:657,959	T 102P 1:695,673	T 126P 1:563,689	T 112P 1:634,057

*T, total, i.e., sum of female and male cases; F, female; M, male; Ratio, male-to-female ratio; P, live prevalence*.

a *No sex strata*.

b *Cells below 5, hence no presentation for any age strata or sex*.

Comparing the patient numbers in the three age groups with the Cochrane-Armitage-trend-test, it becomes apparent that the attending incidence in adolescence and even more in adulthood is significantly lower for epispadias (*p* < 0.001) and exstrophy (*p* < 0.001).

## Discussion

**Figure 1:**
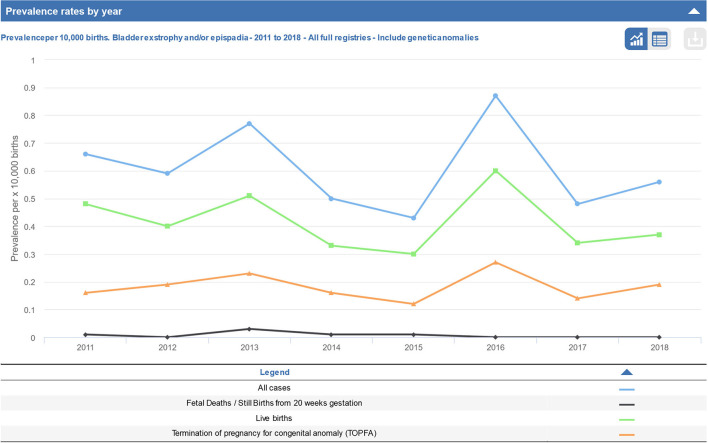
Prevalence rate per year across all registries of bladder exstrophy and/or epispadias from 2011 to 2018 (taken from EUROCAT report).

There is no doubt that epidemiological and clinical research in rare diseases need basic incidence and prevalence data. Due to the lack of a mandatory reporting of congenital anomalies to a nationwide birth registry in many European countries including Germany, only estimations from EUROCAT or German birth registries, the National American Insurance database, or published European epidemiological studies are available for research purposes (5, 10, 12–15). The German EUROCAT-Registry in Saxony-Anhalt which covers approximately 2% of the German population reports for the last decade an incidence for epispadias 1:34,500 (95% CI: 1:15,900; 1:100,000) and for exstrophy 1:29,400 (95% CI: 1:14,500;1:76,900) (6). These results can only be considered as an incomplete and insufficient random sample. The prevalence for EEC reported in Germany was estimated with 6.7 per 100,000 live births (1:14,900). The population-based registries (*n* = 76 members) in EUROCAT are active and regularly contacting birth clinics throughout Europe to transmit data on congenital anomaly cases in their region in a standardized way. EUROCAT indicated the prevalence of bladder exstrophy and epispadias with 4.3 per 100,000 (1:23,300) live births in their last published report in 2017 (7). In all European registries live incidences were between 1:33,300 in 2015 and 1:16,700 in 2016 (cf. Figure 1). The international clearinghouse for birth defects monitoring systems provided an average prevalence rate of 3.3 per 100,000 (1:30,300) for bladder exstrophy and 2.4 per 100,000 (1:41,700) for isolated epispadias (12). A National American Insurance database study (15) showed a significant increase for epispadias from 8.0/100,000 to 11.6/100,000 and a decrease in the birth prevalence of bladder exstrophy from 2.4/100,000 to 1.6/100,000 during the years from 1997 till 2009 (15). This decrease may have occurred as a result of an increase in prenatal pregnancy termination, which was noted in at least 25% after prenatally diagnosed bladder exstrophy cases in the EUROCAT registry (7, 16). However, antenatal diagnosis was made in 3% of male epispadias, 16.4% in bladder exstrophy, and 28% of cloacal exstrophy according to malformation severity (5). From German CURE-Net data we know that only 8.8% of all available participants with classical exstrophies were prenatally diagnosed.

With another method, a prospective survey from the European Society of Pediatric Urology calculated EEC incidence by national reporting through expert centers (5). EEC incidence was calculated for the whole EEC with 1 in 30,000, for male epispadias 1:101,000 live births, 1:1,300,000 for female epispadias, and for isolated bladder exstrophy 1:46,000 (5). The incidence in Germany alone for the year 2010 was rated to be each 1:32,200 for exstrophy (*n* = 21), 1:96,800 for male epispadias (*n* = 7), and 1:677,900 for female epispadias (*n* = 1) (5).

The most valid live prevalence data are listed in Table 4. These data are most valid since all EEC newborns were promptly examined and generally received immediate surgical treatment. According to the presented DIMDI data (2009–2011) the live prevalence for exstrophy was 1:30,675 (in males 1:27,166 and in females 1:35,501). This would mean that 22 exstrophy individuals are born each year in Germany. In 2010 this would have been nearly concordant with the 21 reported exstrophy cases from the Cervellione et al. study (5). Although prenatal exstrophy diagnosis seems to be more often reported than previously, exstrophy prevalence from DIMDI data remained quite stable over the reported years, indirectly suggesting against a significant increase in the rate of prenatal termination for this diagnosis. However, there is only indirect evidence for this statement.

For epispadias, DIMDI prevalence was calculated to be 1:17,142, much higher than previously reported (5). This number corresponds to 39 epispadias of all types each year for the complete German newborn population. The German EUROCAT registry (6) reported an incidence of 1:34,500, with a large confidence interval. In the independent patient registry CURE-Net only a minority of participants had epispadias (14% in both newborns and in the older age cases) compared to exstrophy (8). The majority had the most severe epispadias grade 3 (80% of the newborn and 78% of older age epispadias cases), whereas only a minority of patients had epispadias grade 1 (10% of newborn/13% of older epispadias patients) and epispadias grade 2 (10% of newborn/9% of older age epispadias patients) (8). This report is at odds with the dominant observation that the less severe phenotypes occur most frequently. This is most likely a result of many cases of isolated epispadias, especially grades 1 and 2, being treated in smaller hospitals and so not being reported or included in the previously reported data from excellence centers (5). Additionally, while severe epispadias is associated with risk for incontinence, in up to 75% (10), lower grade epispadias patients are much more likely to be continent. Because of this they do not seek medical care as often and so may not be included and reported in the data from major centers (5) or even specific registries.

The lower male-to-female ratio in adults is likely caused by male-to-female ratio changes in the general population due to higher overall survival of females. Variable male-to-female ratios were previously reported (5, 10, 12–15). Epidemiological studies reported a male-to-female ratio of 2.2:1 for epispadias and a 1.8:1 male predominance for classical bladder exstrophy (13) as well as of 1.4:1 for epispadias and 2.8:1 for exstrophy (14). In CURE-Net, the majority of participants were male (68%), with a male-to-female ratio of 2.2:1 and 2.1:1 in the newborn and older age patient groups, respectively, (8). Taken together, a male predominance in EEC is consistently reported, even in the DIMDI data over several age groups. The incidence of female epispadias, in contrast, is probably underestimated because the condition is seldom diagnosed at birth.

Compared to infants (5.8 per 100,000), older epispadias patients seem to make significantly less use of medical care during childhood. Therefore, their prevalence in the insurance data appears to be decreasing. In the age group from 1 to 17 years, approximately only 18.5 epispadias patients (2.8 per 100,000) were treated per year, whereas about 22 exstrophy patients (3.4 per 100,000) were visited per year (Tables 3, 4). For exstrophy, the prevalence and use of medical care does not significantly change between infants and adolescents. However, in adulthood, even less frequent outpatient treatment per year for epispadias (0.6 per 100,000) and for exstrophy (1.3 per 100,000) is apparent. This is probably due to less medical care in this age group, but it could also generate an impression of a lower live prevalence in older age or lower incidence in former years, as it was already reported for epispadias (15). However, the true reasons for this finding remain unclear.

For cloaca (Q43.7) only a global live prevalence (1:634,057) could be calculated, which is likely biased due to lack of coding in older ages. This could be one reason for the low frequency (*n* < 5), which do not permit further age stratification. Another reason may be low numbers in the smallest age group of infant babies below 1 year of age. If the case number for this smallest group is reduced, the prevalence would be not more than 1:116,566, both far below the otherwise reported prevalence of 1:50,000 (17). This further supports the need for representative reporting among infants.

As already stated, insurance data only provide evidence about live prevalence of the anomaly. Furthermore, limitations of any data collection may have an unpredictable encryption error. Even assuming that all male exstrophy patients below 1 year of age with both diagnosis (Q64.0 and Q64.1) were simultaneously reported, the live incidence for epispadias would still be over the previously reported level: (34 – 11 = 23; hence, at least 1:25,341 or 3.9 per 100,000, if not 5.8 epispadias per 100,000). The reported ICD code Q43.7 includes cloacal exstrophies and other cloacal anomalies to an unknown extent with no possibility to discriminate these entities exactly by this insurance data analysis. From systematic scientific point of view complete insurance data were reported here. Furthermore, DIMDI ensured that each case was encoded once. However, as data seem to be quite consistent over the years, such an error can most probably be excluded. In addition, especially for inpatient treatment, quality assurance by the medical service of the health insurance companies is usually monitored with plausibility check. Possible incorrect or indecisive data are inquired and sent back to the hospitals for further discussion or correction if the accounting could not be proven and was incorrect. Whether this includes internal medical treatment or operations cannot be said. Furthermore, no information is given from DIMDI analysis whether complications with these diagnoses or the diagnosis itself warranted the inpatient hospital stay. Further medical analysis about associated diagnoses or procedures, however, would definitively allow further conclusions. One major benefit of these insurance data is (a) that they have been validated by the insurances to protect from incorrect billing and (b) that they clearly refer to specific individuals even for several visits during 1 year, and hence double counting is prevented. Further, (c) they are quite complete and thus representative for about 71 million people with public health insurances representing about 87% of the German population. Although the data from private insurances were not accessible, no systemic error can be expected in this limited selection, due to the nature of the anomaly. However, as this is a specific German analysis, we believe that this data retrieval methodology may be applied for further research purposes in Germany. Additionally, it could be adjusted to other countries with comparable nationwide and valid insurance data.

Taken together, analyzing DIMDI data sources allows a more precise prevalence estimation of rare diseases such as those within the spectrum of EEC. Furthermore, it provides valuable information about both inpatient and outpatient treatment needs among German patients of all age groups. Detailed additional data including operation procedures, complications, or comorbidities would improve knowledge about the medical needs of individuals with complex and rare anomalies.

## Conclusion

Although any data collection has limitations, these DIMDI insurance data for Germany seem to be quite reliable and complete over the consecutive years 2009–2011. While comparable live prevalence of exstrophy has been previously reported by a different methodological approach, our calculations yielded a higher lifetime prevalence of epispadias at 1:17,142. It is likely that milder epispadias (grade 1 or 2) are insufficiently recorded in the currently available data sources such as European survey, CURE-Net and EUROCAT Registry Malformation Monitoring Saxony-Anhalt, or the personal experience of self-help groups.

For the first time, national coverage data for different age groups were analyzed. Adult or adolescent epispadias and exstrophy patients are less likely to seek medical care. In conclusion, this report offers a first impression of the potential analyses possible using the legally guaranteed access to insurance data in Germany.

## Data Availability Statement

The original contributions presented in the study are included in the article, further inquiries can be directed to the corresponding author.

## Author Contributions

EJ and A-KE had the idea and wrote the manuscript. EJ performed all calculations. NZ and A-KE wrote the proposal to the DIMDI. EJ and HR initiated Network for Systematic Investigation of the Molecular Causes, Clinical Implications and Psychosocial Outcome of Congenital Uro-Rectal Malformations (CURE-Net). All authors read and approved the final manuscript.

## Funding

This work was done in the context of the Network for Systematic Investigation of the Molecular Causes, Clinical Implications and Psychosocial Outcome of Congenital Uro-Rectal Malformations (CURE-Net) and supported by a research Grant (01GM08107) from the German Federal Ministry of Education and Research (Bundesministerium für Bildung und Forschung, BMBF) 2009–2012. Statistical calculations were supported by the German Research Foundation (Deutsche Forschungsgemeinschaft, DFG), funding signs JE681/3-1 (2013–2016), EB521/2-1, and JE681/4-1 (2015–2018). HR was supported by a grant from the DFG (RE 1723/1-1). http://www.cure-net.de.

## Conflict of Interest

The authors declare that the research was conducted in the absence of any commercial or financial relationships that could be construed as a potential conflict of interest. The handling editor declared a past co-authorship with several of the authors HR, NZ, and EJ.

## Publisher's Note

All claims expressed in this article are solely those of the authors and do not necessarily represent those of their affiliated organizations, or those of the publisher, the editors and the reviewers. Any product that may be evaluated in this article, or claim that may be made by its manufacturer, is not guaranteed or endorsed by the publisher.
